# Validation of dual-energy CT-based composition analysis using fresh animal tissues and composition-optimized tissue equivalent samples

**DOI:** 10.1088/1361-6560/ad68bc

**Published:** 2024-08-12

**Authors:** Katharina Niepel, Sebastian Tattenberg, Raanan Marants, Guyue Hu, Thomas Bortfeld, Joost Verburg, Atchar Sudhyadhom, Guillaume Landry, Katia Parodi

**Affiliations:** 1 Department of Medical Physics, Ludwig-Maximilians-Universität München (LMU Munich), Garching, Germany; 2 Department of Radiation Oncology, Massachusetts General Hospital and Harvard Medical School, Boston, United States of America; 3 Department of Radiation Oncology, Brigham and Women’s Hospital, Boston, United States of America; 4 Department of Radiation Oncology, LMU University Hospital, LMU Munich, Munich, Germany; 5 German Cancer Consortium (DKTK), partner site Munich, a partnership between DKFZ and LMU University Hospital Munich, Germany; 6 Bavarian Cancer Research Center (BZKF), Munich, Germany

**Keywords:** dual-energy CT (DECT), proton therapy, prompt gamma spectroscopy, elemental composition

## Abstract

*Objective.* Proton therapy allows for highly conformal dose deposition, but is sensitive to range uncertainties. Several approaches currently under development measure composition-dependent secondary radiation to monitor the delivered proton range *in-vivo*. To fully utilize these methods, an estimate of the elemental composition of the patient’s tissue is often needed. *Approach.* A published dual-energy computed tomography (DECT)-based composition-extraction algorithm was validated against reference compositions obtained with two independent methods. For this purpose, a set of phantoms containing either fresh porcine tissue or tissue-mimicking samples with known, realistic compositions were imaged with a CT scanner at two different energies. Then, the prompt gamma-ray (PG) signal during proton irradiation was measured with a PG detector prototype. The PG workflow used pre-calculated Monte Carlo simulations to obtain an optimized estimate of the sample’s carbon and oxygen contents. The compositions were also assessed with chemical combustion analysis (CCA), and the stopping-power ratio (SPR) was measured with a multi-layer ionization chamber. The DECT images were used to calculate SPR-, density- and elemental composition maps, and to assign voxel-wise compositions from a selection of human tissues. For a more comprehensive set of reference compositions, the original selection was extended by 135 additional tissues, corresponding to spongiosa, high-density bones and low-density tissues. *Results.* The root-mean-square error for the soft tissue carbon and oxygen content was 8.5 wt% and 9.5 wt% relative to the CCA result and 2.1 wt% and 10.3 wt% relative to the PG result. The phosphorous and calcium content were predicted within 0.4 wt% and 1.1 wt% of the CCA results, respectively. The largest discrepancies were encountered in samples whose composition deviated the most from tabulated compositions or that were more inhomogeneous. *Significance.* Overall, DECT-based composition estimations of relevant elements were in equal or better agreement with the ground truth than the established SECT-approach and could contribute to *in-vivo* dose verification measurements.

## 1. Introduction

Proton beams deposit most of their dose in a small region at the end of their range, the well-known Bragg peak, with little dose being delivered to the healthy tissue surrounding the target. This allows for greater dose conformality than photon therapy, but it also makes the treatment more sensitive to range uncertainties, which result from both treatment planning and from changes in the patient’s anatomy and positioning between fractions. Therefore, predicting and monitoring the position of the Bragg peaks inside the patient remains one of the main challenges to fully utilize the potential of proton therapy (Paganetti *et al* 2021).

Several *in-vivo* dose monitoring systems that rely on secondary radiation are currently being studied, such as prompt gamma-ray imaging (PG) (Hueso-Gonzalez *et al* 2018, Krimmer *et al* 2018) and positron emission tomography (PET) (Parodi *et al* 2007b). Since secondary radiation is typically dependent on the elemental composition of the irradiated tissue, in particular on the content of carbon and oxygen, knowledge of the density of these relevant elements within the patient anatomy is critical in order to provide reliable Monte Carlo simulations for these verification methods (Parodi *et al* 2007a, Verburg *et al* 2012). One commonly used approach to derive maps of the patient’s elemental composition is by combining an x-ray computed tomography image acquired at a single energy x-ray energy spectrum (SECT) with a look-up table based on stoichiometric calibration (Schneider *et al* 2000). For this, a CT scanner and a setting-specific calibration based on a set of tissue-mimicking phantom materials are used to create a continuous mapping between the CT numbers in Hounsfield units (HU) of the SECT image and reference compositions and densities. However, this method yields limited accuracy in certain anatomical locations, such as in differentiating between soft tissue and bone marrow (Knopf *et al* 2009) and is specific to a given scanner, setting and choice of calibration phantom materials (Goma *et al* 2018).

Dual-energy CT (DECT) can also be utilized to decrease the range uncertainty of protons (Peters *et al* 2022) by improving the accuracy of stopping power relative to water (SPR) estimation (Niepel *et al* 2021), as well as to provide other tissue properties such as electron and mass density and elemental composition. DECT is therefore increasingly finding use in clinical proton therapy facilities (Wohlfahrt *et al* 2017, Kassaee *et al* 2021). Various DECT-based composition estimation methods have been proposed (Landry *et al* 2013, Hünemohr *et al* 2014, Lalonde and Bouchard 2016, Berndt *et al* 2017). However, the validation of these methods has been limited so far by the unrealistic composition of the studied samples (Landry *et al* 2013), by the lack of a precise ground truth or by the limited number of tissue-mimicking mixtures (Berndt *et al* 2017).

In this study, a published DECT-based method to extract composition information was adapted and benchmarked against both chemical combustion analysis (CCA) and PG-based composition measurements. To this end, samples of finely minced fresh animal tissues mixed with blood as well as mixed samples that mimic the composition and radiological behavior of human tissues (Scholey *et al* 2021, Marants *et al* 2023) were studied.

## 2. Materials and methods

### 2.1. Sample preparation and physical characterization

Two fresh animal tissue samples and four mixed tissue-substitute samples were prepared in two double PMMA boxes with outer dimensions of 22 × 22 × 12 cm^3^ and two single PMMA boxes with outer dimensions of 22 × 12 × 12 cm^3^. The outer walls of the boxes were 10 mm thick and double boxes were separated into two compartments by a 2.5 mm PMMA wall. The lids of the boxes could be screwed onto the boxes and made watertight with a plastic gasket. Details of the preparation are given in the subsequent sections.

#### 2.1.1. Animal tissue phantoms

The two animal tissue phantoms were prepared by filling fresh, chopped porcine brain and liver tissues into the phantom containers and filling up the air pockets with porcine blood. The blood was previously mixed with a small amount of heparin as an anticoagulant. The final samples contained 16 wt% (weight percentage) blood for the brain phantom and 19 wt% blood for the liver phantom. This process was done within 24 h after sacrificing the animals and the phantoms were CT-scanned (see section 2.2) and irradiated for the physical characterization (see section 2.1.3) and PG measurements (see section 2.4) immediately after being prepared, to ensure freshness of the tissues. Three small brain and liver samples of the same batch were used for elemental composition measurements at a specialized microanalytical facility via CCA (see section 2.3). The composition of the resulting phantoms was calculated by combining these measured compositions with the published composition of blood (Woodard and White 1986). Once in their boxes, the samples had dimensions 20 cm (*l*) × 10 cm (*h*) × 10 cm (*w*), with *l* aligned with the beam direction and *w* parallel to the treatment table.

#### 2.1.2. Tissue-mimicking phantoms

Four tissue-mimicking samples representing muscle, adipose, spongiosa and cortical bone tissue were created by mixing deionized water, gelatin, lard, hydroxyapatite powder and sodium dodecyl sulfate, the latter acting as a surfactant to facilitate a homogenous mixing of water and fat. The recipes were optimized to mimic tabulated tissue composition and density (White 1989) as well as x-ray attenuation and water and proton content (Scholey *et al* 2021).

The ground truth compositions of these samples were calculated using the compositions of their constituents. Here the compositions of ultrapure water, hydroxyapatite and sodium dodecyl sulfate was assumed to be chemically pure, whereas the ground truth compositions of gelatin and lard were determined using CCA (see section 2.3).

A sample’s mass density was calculated from weighing the phantom container empty, filled with water, and filled with the sample using a high-precision scale. The volume was determined by subtracting the weight of the container from the water-filled container and adding a correction to account for any large air pockets that may have been present at the top of the samples (see figures 3(A) and (B)). Once in their boxes, the samples had dimensions 20 cm (*l*) × 10 cm (*h*) × 10 cm (*w*).

#### 2.1.3. Physical characterization of samples

**Figure 1.**
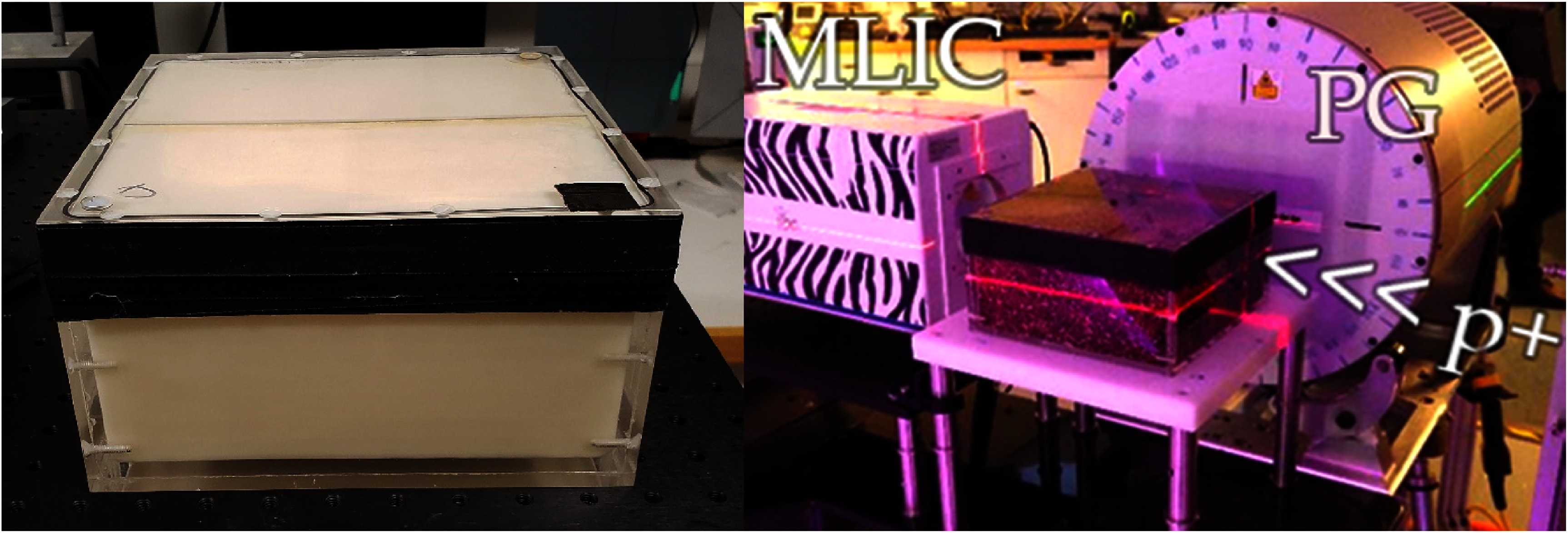
(left) Phantom box containing the mixed samples mimicking spongiosa and adipose and (right) phantom box containing fresh animal tissue samples placed in the complete irradiation setup. The samples were clamped on an adjustable platform to ensure a reproducible position, and the height was adjusted so that the protons (p+) coming from the right shoot through the center of each sample before reaching the collecting electrode of the multi-layer ionization chamber (MLIC). The prompt gamma-ray (PG, see section 2.4) detector is positioned perpendicular to the beam direction. Figure adapted with permission from the accompanying study by Tattenberg *et al* (2022). Adapted from Tattenberg *et al* (2022). © 2022 Institute of Physics and Engineering in Medicine. All rights reserved.

The ground truth stopping power of all ex-vivo and tissue-mimicking samples was determined using a multi-layer ionization chamber (MLIC) (Zebra, IBA Dosimetry, Schwarzenbruck, Germany). MLIC measurements of SPR were performed utilizing a single isocentric proton beam with an energy of 223.6 MeV and a beam current of 2 nA (see figure 1, right). The initial proton energy corresponded to a spot size of 8.0 mm (sigma) in air at isocenter, and Monte Carlo simulations performed with the code FLUKA estimated the spot size just downstream of the samples to be smaller than or equal to 10.6 mm (sigma) for all samples (Battistoni *et al* 2016). The collecting electrode of the MLIC has a diameter of 25 mm, and positioning placed the isocentric proton beam in the center of the MLIC’s collecting electrode to assure sufficient coverage of the proton beam. For SPR determination, repeated range measurements of the phantom boxes filled with the sample, water and air were taken and the distal positions at 80% of the maximum Bragg peak for each box filling were used to calculate the sample’s SPR according to (Niepel *et al* 2021):

\begin{align*}{\text{SP}}{{\text{R}}_{{\mathbf{sample}}}} = \frac{{{{R}}_{{\mathbf{sample}}}^{{80}} - {{R}}_{{\mathbf{air}}}^{{80}}}}{{{{R}}_{{\mathbf{water}}}^{{80}} - {{R}}_{{\mathbf{air}}}^{{80}}}}\left( {{1} - {{\,}}{\text{SP}}{{\text{R}}_{{\mathbf{air}}}}} \right) + {\text{SP}}{{\text{R}}_{{\mathbf{air}}}}\end{align*}

with the SPR of dry air SPR_air_ for 223.6 MeV taken from the PSTAR database of the National Institute of Standards and Technology (Berger 2017).

### 2.2. DECT imaging and data analysis

#### 2.2.1. DECT scans

All phantoms were consecutively scanned at a conventional CT scanner using a head protocol (GE Medical Systems Discovery CT590 RT, General Electric Company, USA) with a lower energy of 80 kV (500 mA tube current, 111 mAs exposure, 54.77 mGy CTDI_vol_) and a higher energy of 140 kV (250 mA tube current, 55 mAs exposure, 107.32 mGy CTDI_vol_). The scans were acquired with a pitch of 0.56, a single collimation width of 0.625 mm and a total width of 10 mm, and the CTDI_vol_ was reported for the head CTDI phantom. The scan field of view was 500 mm and the spacing between slices as well as the slice thickness were 1.25 mm. The image pairs were reconstructed using the 100% adaptive statistical iterative reconstruction (ASiR) with the vendor’s standard reconstruction kernel on a 0.976 mm × 0.976 mm × 1.25 mm grid and analyzed as a DECT image.

The cortical bone-mimicking sample was filled in a single PMMA container (outer dimensions of 22 (*l*) × 12 (*h*) × 12 (*w*) cm^3^, sample dimensions 20 (*l*) × 10 (*h*) × 10 (*w*) cm^3^) to avoid artefacts caused by its high attenuation coefficient. However, in the original scan the box was positioned with its long axis (*l*) orthogonal to the CT rotation axis, resulting in significant beam hardening artefacts. We therefore decided to re-fill this sample into the phantom, position it parallel to the rotation axis of the scanner and repeat the CT scan for this sample only.

#### 2.2.2. Calibration and validation imaging phantoms

To calibrate the DECT conversion algorithm to the specific scanner and scan protocol, a set of twelve tissue-equivalent inserts (Gammex RMI, Sun Nuclear Corporation, Melbourne, FL, USA) were stacked 4 at a time in the central bore of a cylindrical PMMA phantom of diameter 13 cm and scanned using the same protocols as the mixed and animal samples (see previous section). Additionally, a second cylindrical PMMA phantom of diameter 15 cm containing six tissue-equivalent inserts (CIRS, Computerized Imaging Reference Systems Inc. Norfolk, USA) was scanned to evaluate the accuracy of the DECT conversion algorithm for this setup. The inserts of both phantoms as well as their physical properties are given in table 1 of (Hu *et al* 2022) and in the supplementary material to this article.

Both the calibration and validation inserts had known composition, mass- and electron-density (from manufacturer data) as well as SPR determined in previous measurements using a variable water column encompassed by two ionization chambers (PTW peakfinder, PTW, Freiburg, Germany) (Niepel *et al* 2021). Mean CT numbers within cylindrical regions of interest (ROIs) of all calibration phantom inserts were calculated for both tube potentials to fit the specific DECT conversion parameters (see next section) for this specific scanner and scan parameters. This process was repeated with the validation phantom inserts, to assess the accuracy of the fits.

#### 2.2.3. CT conversion algorithm

**Figure 2.**
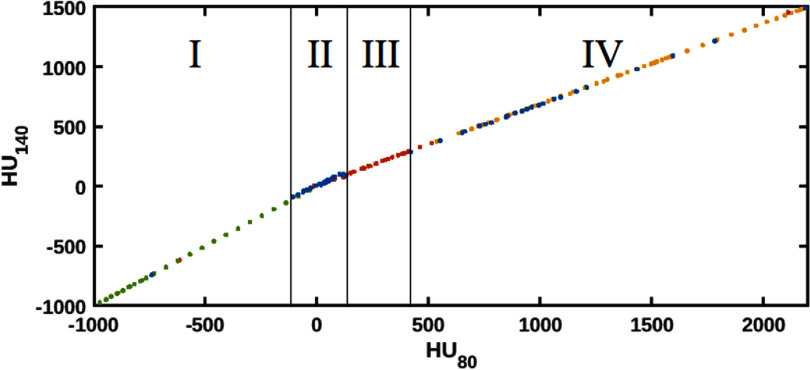
Expected CT numbers at 80 kV (HU_80_) and 140 kV (HU_140_) for human tissue compositions from Woodard and White (1986), White *et al* (1987) (blue) and for additional spongiosa and soft tissue compositions from ICRP report 110 (Menzel *et al* 2009) (red) that fill the gap (III) between soft tissues (II) and bone tissues (IV). To ensure that the full CT number range is densely covered, additional reference compositions have been added to fill the low CT number area (I) with low-density soft tissues (green) and the high CT number area (IV) with additional bone tissues (yellow).

Elemental composition maps were obtained by assigning reference compositions out of a comprehensive set of published human tissue data. This was done by calculating predicted CT numbers for each reference tissue for both CT scan protocols and assigning the composition of the closest reference point in the DECT space to each voxel without interpolation (Berndt *et al* 2017). The predicted CT numbers were obtained from the stoichiometric method (Schneider *et al* 2000) after fitting the parameters *k*_1_ and *k*_2_ to the scans of the calibration phantom at each energy, as outlined in Berndt *et al* (2017). The results of that fitting procedure are reported in the supplementary material to this article. The dataset used in the original publication (White *et al* 1989) uses averaged values for different bone sites rather than reflecting the inner structure of larger bones, resulting in a large gap between expected CT values for soft and bony tissues (see figure 2, area III). In this study, various spongiosa tissues based on the ICRP reference human (Menzel *et al* 2009) were added to the set to fill this gap and obtain a better reflection of the structure of bony tissues. The low CT number region between air, lung and adipose tissue contained only very few reference points (see figure 2, area I). However, partial volume averaging for voxels at the interface of lung and air, for example, could result in such low CT numbers. To account for this, additional low-density tissues were created as combinations of lung tissue and air properties with varying volumetric proportions. Additionally, the sparse selection of reference tissues in the high CT number (figure 2, area IV) range were filled in using additional bone reference tissues generated as described in the supplementary material of Berndt *et al* (2017). The resulting selection of reference points is represented in figure 2. The materials in figure 2 are represented as points without overlap. In the case of materials which are in close proximity to each other, the reference tissue which is closest to the measurement is selected.

The DECT images were converted into SPR maps using a method published by Saito and Sagara (2017a, b). This method relies on parametrizations of the relative electron density $\widehat {\,{\rho _e}}$ as a weighted sum of high- and low-energy CT numbers, and of the effective atomic number Z_eff_ as a function of $\widehat {\,{\rho _e}}$ and the low-energy CT image. The effective atomic number was then converted into the ionization potential *I*. For the full formulations please refer to equations (1)–(4) in Hu *et al* (2022) and to the supplementary material to this article. These properties were then combined to obtain the relative stopping power according to:

\begin{equation*}{\text{SPR}} = \widehat {{{\,}}{{{\rho }}_{{e}}}}{{\,}}\frac{{\ln \frac{{2{{{m}}_{\mathbf{e}}}{{{c}}^2}{{{\beta }}^2}}}{{{{I}}\left( {1 - {{{\beta }}^2}} \right)}} - {{{\beta }}^2}}}{{\ln \frac{{2{{{m}}_{{e}}}{{{c}}^2}{{{\beta }}^2}}}{{{{{I}}_{{w}}}\left( {1 - {{{\beta }}^2}} \right)}} - {{\,}}{{{\beta }}^2}}}\end{equation*}

with electron rest mass *m*_e_ and speed of light *c*. In this work, we assumed the ionization potential of water *I*_w_ = 78 eV (Ziegler 1999) and relative proton velocity *β* = 0.482, corresponding to an intermediate proton energy of 100 MeV. DECT-based mass density maps were calculated by combining electron densities of Saito and Sagara’s approach with the assigned elemental compositions.

In addition to the SECT-based look-up table conversion used in the treatment planning step of the PG workflow (see section 2.4), an implementation of the stoichiometric conversion method described by Schneider *et al* (2000) was performed using the 140 kV scan and the calibration phantom. This approach uses linear interpolation within intervals of published densities and compositions as functions of the stoichiometrically predicted CT numbers of the corresponding tissues. In this work, the calibrated implementation of Schneider’s method will be labeled with ‘SECT’ and the result of the look-up table used for treatment planning will be referred to as ‘HLUT’.

### 2.3. CCA

The elemental composition of the porcine samples, gelatin and porcine lard was analyzed at the Berkeley College of Chemistry Microanalytical Facility (Berkeley, CA, USA). For this purpose, three brain and three liver samples from the same batch as the tissue phantom were randomly selected from the batch and slowly dehydrated over the course of 12 h. During this process the samples were regularly weighted, and once the mass stopped changing, they were assumed to be fully dried, and the dry components were grinded into a fine homogeneous powder, sealed in small plastic containers and shipped to UC Berkeley for CHNS(O) combustion analysis (Li and Brimmer 2004). These dry components made up about 13 wt% of the brain samples and 21 wt% of the liver samples, respectively.

The values of the three samples were averaged, and the standard deviation calculated in order to assess how representative these samples were of the entire batch. To derive the composition of the wet tissues pre-dehydration, the lost weight was assumed to be pure water (H_2_O). The compositions of the finalized sample mixtures are summarized in table 1.

**Table 1.** Mean and standard deviation of CT numbers for the low (HU_80_)- and high-energy (HU_140_) CT scans within ROI_SPR_, which intersects the proton beam path during the MLIC measurement, as well as corresponding CT-based stopping power ratio (SPR) and mass density in g cm^−3^ (${\rho _m})$ values calculated using the high-energy scan (SECT) or both scans (DECT) along with ground truth SPRs and mass densities obtained via measurements of the samples. The multi-layer ionization chamber (MLIC) uncertainty was estimated at 0.5%.

	HU_80_	HU_140_	SPR^DECT^	$\rho _m^{{\text{DECT}}}$	$\rho _m^{{\text{SECT}}}$	SPR^MLIC^	$\rho _m^{{\text{measured}}}$
Muscle	51 ± 5	54 ± 3	1.06 ± 0.01	1.05 ± 0.01	1.066 ± 0.004	1.055 ± 0.05	1.062 ± 0.001
Adipose	−125 ± 23	−92 ± 25	0.95 ± 0.03	0.92 ± 0.03	0.94 ± 0.02	0.964 ± 0.05	0.937 ± 0.001
Spongiosa	195 ± 41	126 ± 28	1.05 ± 0.03	1.06 ± 0.03	1.09 ± 0.02	1.064 ± 0.05	1.082 ± 0.001
Cortical	1355 ± 77	879 ± 59	1.35 ± 0.04	1.47 ± 0.05	1.52 ± 0.05	1.320 ± 0.07	1.404 ± 0.001
Brain	22 ± 38	19 ± 42	1.02 ± 0.05	1.01 ± 0.05	1.03 ± 0.04	1.014 ± 0.05	1.009 ± 0.001
Liver	70 ± 9	66 ± 9	1.06 ± 0.02	1.06 ± 0.01	1.08 ± 0.01	1.061 ± 0.05	1.062 ± 0.001

### 2.4. Prompt gamma-ray analysis

The proton range in and elemental densities of the fresh tissue samples and tissue-mimicking samples were independently determined with prompt gamma-ray (PG) spectroscopy (Tattenberg *et al* 2022). The phantoms were positioned in the beam line of a clinical proton irradiation room using orthogonal x-ray imaging with a 1 mm accuracy (see figure 1). A 5 × 5 × 5 cm^3^ target region at a water-equivalent depth of 12.5–17.5 cm in the sample phantom was irradiated with a proton beam with energies between 132.7 MeV and 161.6 MeV (153.7 MeV and 191.3 MeV in the case of the cortical bone sample), delivering a dose of 1 Gy (RBE). A prototype prompt gamma-ray spectroscopy detector (Hueso-Gonzalez *et al* 2018) was placed orthogonally to the proton beam direction upstream of the expected depth of the Bragg peak, where PG cross-sections are the highest, to detect the gamma-ray emission caused by the nuclear interactions of incident protons with the samples (see figure 1).

According to the PG range monitoring workflow, a first estimation of a phantom density and composition map was made using the conversion by Schneider *et al* (table 6 in Schneider *et al* 2000) based on the 140 kV scan, as is done clinically. Expected gamma-ray emissions for different range error scenarios were predicted using Monte Carlo simulations based on these properties (see Hueso-Gonzalez *et al* 2018, Tattenberg *et al* 2022). Differences in range as well as carbon and oxygen density relative to the treatment plan were then determined by fitting the expected PG signal to the measured gamma-ray emissions for each pencil-beam spot. The elemental densities were converted to weight proportions using the phantom density maps to allow for better comparison with the DECT- and CCA-based approaches of sections 2.2 and 2.3, respectively. A more detailed description of the PG setup and analytical workflow is found in the accompanying study by Tattenberg *et al* (2022).

### 2.5. ROI for analysis

Since the investigated areas of the samples differed between the MLIC and PG measurements and combustion analysis, three different ROIs were defined for data evaluation: (1) A cylindrical ROI with a diameter of 4 cm along the center of the sample, which corresponds to the proton beam path during the MLIC measurements and is therefore used for the SPR analysis (ROI_SPR_), and two rectangular ROIs which correspond to (2) the 5 × 5 × 5 cm^3^ target region (ROI_target_) that was irradiated during the PG measurement and (3) the entire sample volume (ROI_sample_). Sample homogeneity was evaluated via a comparison between the values for ROI_sample_ and ROI_target_. In addition, data evaluation was repeated for ROI_SPR_ and ROI_target_ shifted in all dimensions by different distances and compared to the results for the original ROIs to assess the uncertainty introduced by sample inhomogeneity and possible positioning errors (details in the supplementary material to this article).

For the CT-based composition analyses, the relative weight proportion ${w\,^X}\,$ of element *x* was calculated as the mean composition within ROI_target_, weighted by the mass density $\rho _i^m$ for the *i*th voxel according to:

\begin{equation*}{{{w\,}}^{{X}}} = {{\,}}\frac{{{{\mathop \sum \nolimits}}_{{i}}{{w\,}}_{{i}}^{{X}}{{\rho }}_{{i}}^{{m}}}}{{{{\mathop \sum \nolimits}}_{{i}}{{\rho }}_{{i}}^{{m}}}}.\end{equation*}

In addition to ${w^C}$ and ${w^O}$, relative proportions of calcium (${w^{Ca}}$) and phosphorous (${w\,^P}$) were derived for bony tissues since they are particularly important when characterizing such high-density tissues.

## 3. Results

### 3.1. Sample homogeneity

**Figure 3.**
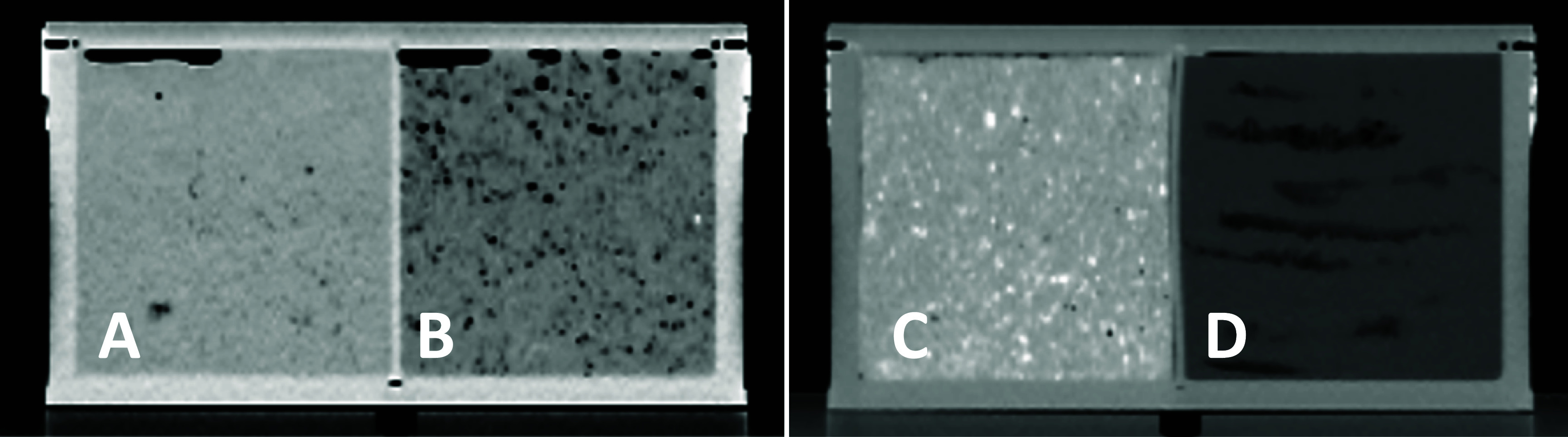
(left) Low-energy CT images of liver (A) and brain (B) (window = 400, level = 0) and (right) spongiosa- (C) and adipose-mimicking (D) samples (window = 700, level = 0). The brain sample exhibits inhomogeneities due to microbubbles of air.

For CCA, the elemental concentrations were very similar between the different samples of the same tissue, with mean elemental composition variations of only 0.3 wt% among brain samples and 0.2 wt% among liver samples. We are therefore confident that the selected probes are reflective of the overall mean composition of the solid porcine tissues. In the assembled fresh tissue phantoms containing solid tissue and blood, the liver sample showed good homogeneity in the CT scan, but the brain sample exhibited significant inhomogeneities in the form of small air bubbles throughout the sample that were not resolved through filling with blood (see figure 3). This resulted in an increased spread in the CT number pairs, with standard deviations of 47 HU and 51 HU for the low- and high-energy scan, respectively, compared to only 9 HU and 10 HU for the liver sample. Of the mixed samples, muscle was very homogenous whereas the adipose tissue-mimicking sample exhibited a layer structure resulting from the manufacturing process and the two bony samples suffered from slight clumping. The mean CT numbers and their standard deviations within ROI_SPR_, which serve as a measure of homogeneity, are summarized in table 1. Table 2 summarizes the DECT-based mean elemental compositions and their standard deviations within ROI_target_ and ROI_sample_.

**Table 2.** Mean and standard deviation of DECT-based elemental compositions in rectangular regions of interest representing the whole sample area (ROI_sample_) and the area irradiated during prompt gamma-ray (PG) measurements (ROI_target_).

	ROI_sample_				ROI_target_			
	w^C^/%	w^O^/%	w^Ca^/%	w^P^/%	w^C^/%	w^O^/%	w^Ca^/%	w^P^/%
Muscle	16.0 ± 3.5	69.0 ± 3.7	0.0 ± 0.1	0.2 ± 0.1	16.2 ± 3.3	68.7 ± 3.5	0.0 ± 0.1	0.2 ± 0.1
Adipose	67.8 ± 3.5	20.1 ± 3.9	0.0 ± 0.0	0.0 ± 0.0	68.1 ± 1.9	19.8 ± 0.6	0.0 ± 0.0	0.0 ± 0.0
Spongiosa	37.6 ± 4.9	44.8 ± 5.0	3.1 ± 0.8	1.6 ± 0.4	37.7 ± 4.5	44.6 ± 4.6	3.2 ± 0.8	1.6 ± 0.4
Cortical	25.1 ± 3.0	41.5 ± 4.2	16.5 ± 1.5	7.6 ± 0.7	24.7 ± 2.8	41.6 ± 4.0	16.9 ± 1.2	7.8 ± 0.6
Brain	17.6 ± 14.7	68.7 ± 17.3	0.0 ± 0.1	0.1 ± 0.1	16.8 ± 13.9	69.4 ± 16.1	0.0 ± 0.1	0.1 ± 0.1
Liver	18.9 ± 10.1	66.1 ± 10.7	0.2 ± 0.5	0.3 ± 0.2	20.2 ± 10.4	64.6 ± 11.0	0.3 ± 0.5	0.3 ± 0.2

### 3.2. Stopping power and mass density

Table 1 summarizes the relative stopping powers based on the ionization chamber measurements (SPR^MLIC^) and the converted DECT scans (SPR^DECT^). Mean DECT-based stopping powers and mass densities based on dual- and single-energy CT ($\rho _m^{{\text{DECT}}}$ and $\rho _m^{{\text{SECT}}}$) values were calculated within a cylindrical ROI with a diameter of 4 cm and a length of 20 cm located in the center of the samples, corresponding to the proton beam path during the MLIC measurements. The measured values for both SPR and mass density remained within one standard deviation (of the distribution of values in ROI_SPR_) of the mean values predicted based on the DECT scans.

The overall root-mean-square error (RMSE) of the DECT-based SPR was 0.5% for soft and 1.9% for bony tissues. The mass density also showed good overall agreement, with an RMSE of 0.7% and 1.2% for soft tissues in case of DECT and SECT, respectively, but larger deviations of 3.2% and 6.1% for the two bony tissues, likely due to the deviations between predicted and actual composition for these samples (see section 3.3).

### 3.3. Elemental composition

The ground truth elemental compositions obtained by combustion analysis are summarized in table 3, along with the confidence interval resulting from the uncertainties of the sample mixing and the combustion measurements.

**Table 3.** Full ground truth elemental compositions of mixed tissue substitutes and fresh animal tissues in percentages of total weight, based on combustion analysis of their constituents. The overall uncertainties of the elemental compositions were calculated by combining the uncertainty of the combustion analysis and the weighting uncertainty of the mixture preparation.

	w^H^/%	w^C/^%	w^N/^%	w^O^/%	w^P^/%	w^S^/%	w^Ca^/%	w^Na^/%
Muscle	10.37 ± 0.04	12.29 ± 0.33	2.98 ± 0.04	74.20 ± 0.38	0.00 ± 0.00	0.14 ± 0.02	0.00 ± 0.00	0.02 ± 0.00
Adipose	12.30 ± 0.05	77.99 ± 0.03	0.00 ± 0.00	9.72 ± 0.03	0.00 ± 0.00	0.00 ± 0.00	0.00 ± 0.00	0.00 ± 0.00
Spongiosa	9.90 ± 0.05	42.55 ± 0.21	1.76 ± 0.03	38.74 ± 0.25	2.13 ± 0.00	0.21 ± 0.01	4.60 ± 0.00	0.11 ± 0.00
Cortical	6.74 ± 0.00	0.00 ± 0.00	0.00 ± 0.00	69.61 ± 0.01	7.49 ± 0.00	0.00 ± 0.00	16.16 ± 0.00	0.00 ± 0.00
Brain	10.96 ± 0.13	5.99 ± 0.52	0.73 ± 0.43	82.27 ± 0.21	0.00 ± 0.00	0.05 ± 0.02	0.00 ± 0.00	0.00 ± 0.00
Liver	10.63 ± 0.05	8.51 ± 0.17	1.94 ± 0.30	78.80 ± 0.25	0.00 ± 0.00	0.11 ± 0.01	0.00 ± 0.00	0.00 ± 0.00

The SECT results for carbon and oxygen are summarized in table 4. For the sake of a fairer comparison, both the compositions based on the Hounsfield unit look-up table (HLUT) used in the treatment planning software and a stoichiometric calibration adjusted to the scanner are listed. For soft tissues, the RMSE of carbon was 11.7 percentage points for both the treatment planning software and Schneider implementation, and 12.2 and 12.1 percentage points in case of oxygen, respectively.

**Table 4.** Elemental weight of carbon and oxygen derived by SECT conversion using the HLUT implemented in the treatment planning software of the prompt gamma-ray spectroscopy workflow and a best-effort implementation of the algorithm by Schneider *et al* [2000] along with the corresponding standard deviations within the region of interest.

	HLUT		SECT	
	w^C^/%	w^O^/%	w^C^/%	w^O^/%
Muscle	13.4 ± 0.1	72.3 ± 0.1	13.6 ± 0.8	71.9 ± 1.0
Adipose	58.5 ± 14.3	29.0 ± 13.7	57.4 ± 20.8	30.0 ± 18.7
Spongiosa	34.8 ± 12.4	47.1 ± 13.4	43.8 ± 7.1	37.5 ± 7.4
Cortical	26.7 ± 1.6	40.5 ± 1.5	26.1 ± 1.8	40.7 ± 2.9
Brain	18.9 ± 11.1	66.9 ± 10.7	17.2 ± 11.8	66.2 ± 13.9
Liver	13.8 ± 12.4	72.2 ± 1.8	16.7 ± 1.9	68.5 ± 2.1

**Figure 4.**
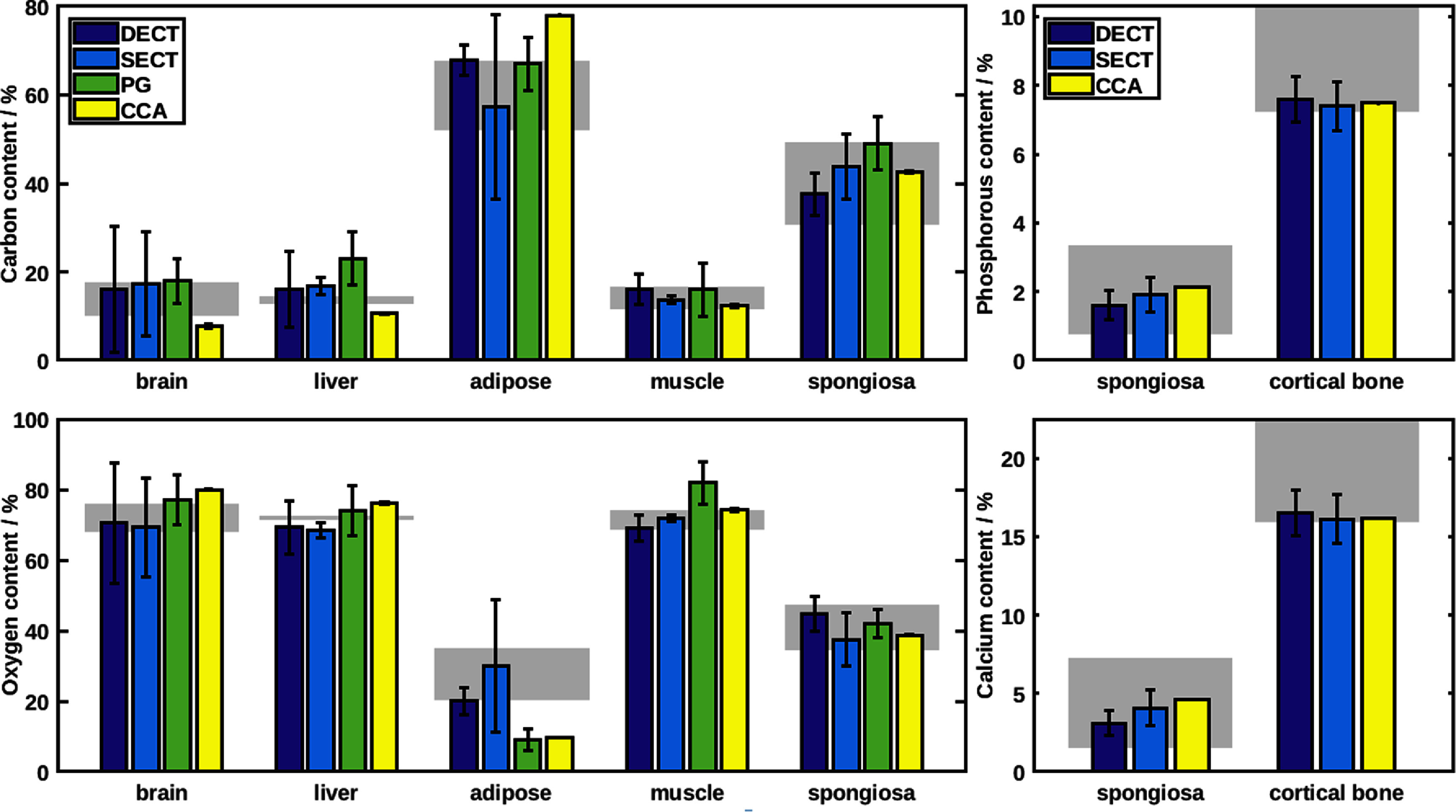
Weight percentile content of carbon, oxygen, phosphorus and calcium for tissue samples predicted using dual energy CT (DECT), prompt gamma-ray spectroscopy (PG), chemical combustion analysis (CCA) and best-effort single energy CT (SECT). The black lines span standard deviations within a square region of interest (ROI) corresponding to the sample area, between proton pencil-beam spots (for PG), or among the investigated samples (in case of combustion analysis). The grey boxes illustrate the ranges of compositions expected from the corresponding tabulated human tissues.

The RMSE between DECT- and PG-based soft tissue compositions was 3.6 wt% and 9.4 wt% for carbon and oxygen, respectively. The RMSE between DECT and CCA of soft tissues yielded slightly larger discrepancies of 7.4 wt% for carbon and 8.2 wt% for oxygen. The RMSE between calibrated SECT conversion and CCA of soft tissues was 11.8 wt% and 12.1 wt% for carbon and oxygen, respectively. The chemical concentrations of phosphorous and calcium was predicted within an RMSE of 0.4 wt% and 1.1 wt% by DECT with respect to CCA. The SECT conversion adjusting the stoichiometric calibration to the scanner had an RMSE of 0.2 wt% and 0.4 wt% when compared to CCA for calcium and phosphorous, respectively. The compositions obtained with all four methods are summarized in figure 4. The PG analysis yielded estimates of only carbon and oxygen content for the irradiated area, as these two elements are the most abundant in human tissue and have the largest influence on emitted gamma radiation. The mean and standard deviation were obtained by averaging over all pencil beams. A full discussion of the PG result can be found in Tattenberg *et al* (2022).

## 4. Discussion

### 4.1. Sample homogeneity and noise

The homogeneity of the tissue and tissue-mimicking samples varied greatly, with the CT images of the porcine liver and the muscle-mimicking sample being highly homogeneous while the images of the spongiosa and cortical bone samples suffered from both clumping in the samples themselves and beam hardening effects with respect to x-ray imaging. The worst inhomogeneity was found in the form of small air bubbles within the porcine brain sample, resulting in the highest standard deviation of CT numbers within the ROI among all samples.

The cortical bone sample was originally scanned with its long axis perpendicular to the CT scanner’s rotational axis, as this was the position needed for the CT used for treatment planning. This positioning resulted in significant beam hardening effects due to the high attenuation of this sample. To remedy this, part of the cortical bone sample was filled back into the phantom box after the measurement and scanned again in a longitudinal position within the CT scanner. In the new orientation, the x-ray paths through the phantoms were much shorter and the scan suffered only minor beam hardening artifacts, but the area investigated in the original prompt gamma-ray measurement was no longer entirely covered by the remaining sample. We opted to use the DECT images of the refilled sample with a smaller region of interest regardless, and all values given in this paper reflect this. Since no cortical bone areas of the thickness of the sample can be found in the human body, this difficulty is not expected to occur in clinical practice.

The elemental contents within the irradiated area and the entire sample showed no significant difference in either mean value or variation. The mean values of the entire samples were therefore used for further comparisons, in accordance with combustion analysis, which likewise aimed to reflect the entire sample.

### 4.2. Stopping power and mass density

As in previous studies of DECT conversion algorithms, the stopping power was predicted with excellent agreement for the soft tissues, with an RMSE of 0.5% and only slightly lower agreement of 1.9% in case of the bony tissues. Here the RMSE for the fresh tissues was even lower than for the soft tissue-mimicking samples, with a deviation of only 0.2% compared to 0.5%, possibly due to the more human tissue-like compositions of these samples. This is consistent with a previous study (Niepel *et al* 2021) in which a selection of six soft tissue samples also yielded a 0.5% RMSE for DECT, as well as a seminal work on the topic (Mohler *et al* 2018).

The mass density was calculated based on electron density and assigned composition in the DECT conversion. It was therefore impacted by the less realistic composition of some tissue-mimicking samples, with the largest deviations occurring for the two bony tissues. The SECT-to-density conversion remained robust to the lack of a scanner-specific calibration, with the non-calibrated HLUT (using table 6 from Schneider *et al* 2000 directly) performing just as well as the calibrated conversion (redoing the table with scanner-specific fits) but neither reaching the accuracy of the DECT-based mass density estimation.

The SPR errors of the DECT-based estimations were at most 1.5% (for adipose) or lower (0.8% on average), indicating that the use of samples of larger dimension than the calibration phantom (15 cm diameter cylinder vs 12 cm × 22 cm boxes) did not have a large impact on our results. A systematic evaluation of the effect of phantom sizes is planned for the future.

### 4.3. Elemental composition

Although not too critical for the accuracy of dose calculations, knowledge of the elemental composition of human tissue can impact *in-vivo* monitoring workflows relying on the spatial distribution of physical emissions such as PET or prompt gamma-ray imaging. This especially applies to the most relevant components in tissue, which are carbon and oxygen.

In this study, carbon and oxygen content of soft tissues and spongiosa were determined with very good agreement by the DECT conversion. The largest discrepancies occurred in the brain sample, likely due to the significant inhomogeneities caused by small air bubbles, as well as in the adipose-mimicking sample, for which the largest discrepancies between sample composition and tabulated human tissue composition were observed.

For the bony samples there was very good agreement of the expected calcium and phosphorous content for both single- and dual-energy CT conversions, with a slight advantage for the SECT method. The cortical bone substitute sample was designed to mainly mimic calcium and phosphorous content of human bone, at the expense of a realistic carbon and oxygen content. As could be expected, only these concentrations were predicted with good accuracy.

#### 4.3.1. Comparison to SECT

The DECT-based composition derivations yielded significant improvements over the SECT approach, with an RMSE of 7.4 and 8.2 percentage points for carbon for oxygen, respectively, compared to the RMSE of 11.8 and 12.1 percentage points obtained with the SECT approach.

Since the particular PG analysis workflow discussed in this work corrects the planning CT-based estimate of the elemental concentration based on the measurement, it is quite robust to minor inaccuracies in the input. The HLUT of the original publication was therefore used during treatment planning, without adjusting to the specific scan settings. The adjustment of the SECT conversion to the scanner via a calibration did not yield a significant change in the overall composition accuracy within the ROIs but did result in a slightly larger standard deviation. Since the studied samples were not fully homogeneous, it is unclear whether this reflects actual variation of the samples or a higher sensitivity to noise due to the larger number of intervals.

The cortical bone sample’s SECT-based composition estimation suffered from similar errors as it did for the DECT-based conversion. This was likely caused by its unrealistic carbon and oxygen content and the larger error in the density estimation for this sample. No significant improvement was observed subsequently to expansion of the base tissue selection, as could be expected since the SECT analysis already relies on interpolating a small number of tissue types across large CT number intervals.

#### 4.3.2. Comparison to PG

The PG analysis relies on a non-linear optimization of the elemental density rather than composition. For the sake of this study, the elemental weights were obtained by dividing elemental densities by the SECT-derived mass density that was also used as input in the optimization, in order to allow for an easier comparison with the DECT, SECT and CCA results. Therefore, it is impossible to differentiate between discrepancies resulting from the mass density or the PG optimization.

The DECT-based carbon content was closer to the prompt gamma-ray spectroscopy result than the CCA result for four out of five soft tissue samples, and for three out of the five samples in the case of oxygen. This could indicate inhomogeneity in the studied samples, for example a varying mix of dry tissue and blood in case of the porcine tissue or clumping for the tissue-mimicking samples, which impact the accuracy of the ground truth for the investigated region of interest. Using CCA as ground truth, DECT yielded a more accurate estimation of the carbon content, while PG yielded a better estimation of the oxygen content. The PG-based carbon content was significantly overestimated for all soft tissues except the adipose-mimicking sample, resulting in very high combined contents of carbon and oxygen between 95% and up to 98% of the total tissue mass for the brain, liver and muscle sample. For comparison, the published human tissue compositions never have more than 88.3% combined mass for these two elements (White *et al* 1989), and the CCA analysis never showed more than 87.7%. This could reflect either an overestimation of the elemental densities for these two elements or an underestimation of the mass density by SECT. Adding a boundary condition based on human tissue data in combination with a more reliable mass density input based on DECT for this combined elemental content could improve the estimate of the carbon and oxygen density and potentially yield a better estimation of the proton range.

Relative to the results from combustion analysis, the PG-based refinement of the average starting composition based on SECT corrected the mean carbon content in all soft tissues except for the liver sample and the mean oxygen content in all soft tissues but muscle.

#### 4.3.3. Influence of tissue basis selection

The extension of the tissue basis selection for the DECT-based analysis resulted in a significant improvement of the composition estimation for the spongiosa-mimicking sample and only minor changes in the composition of the other samples. Table 5 summarizes the impact of the expanded selection of input tissues on the spongiosa sample, compared to a basis of only the ICRU tissues [White 1989 ]. The addition of low-density tissues representing voxels affected by partial volume mixing of air and tissue only resulted in minor improvements for the composition of samples with air bubbles (such as the porcine brain sample), since its composition was already at a very low weighting by the correctly identified low electron density.

**Table 5.** Mean and standard deviation of the DECT-derived elemental composition of the spongiosa-mimicking sample obtained with the tissue selection (Berndt *et al* 2017)] of the original publication (White 1989) of the conversion algorithm (‘ICRU basis’) and the expanded basis which included more bony examples and mixtures of other tissues as well as the ground truth, which was obtained via combustion analysis.

	ICRU basis	Expanded basis	Ground truth
wC/%	12.0 ± 2.6	37.6 ± 4.9	42.6 ± 0.2
wO/%	74.1 ± 3.9	44.8 ± 5.0	38.7 ± 0.3
wP/%	0. ± 0.7	1.6 ± 0.4	2.1 ± 0.0
wCa/%	0.1 ± 0.9	3.1 ± 0.8	4.6 ± 0.0

Since the additional tissue base compositions either are or closely resemble combinations of already included tissues and the stoichiometric analysis relies on linear combinations of a few selected tissues, this extension is not expected to affect the SECT conversion.

## 5. Conclusion

We refined and validated a published DECT conversion algorithm and a well-established SECT conversion against two independent reference composition measurements in the form of prompt gamma-ray spectroscopy and CHNS(O) combustion analysis. The influence of the basis of human tissue compositions on the composition prediction by DECT was highlighted, especially in the case of samples that deviated from expected compositions. Since all DECT-to-composition conversion algorithms published to date include this input in some form and most relied on ICRU report 44 from 1989, a more careful look at the selection of expected compositions is advisable, and the inclusion of more spongiosa tissue types in this paper led to improvements. Further studies could include more refined tissue bases including varying elemental compositions in bodies of children and seniors or in cancer tissue, as well as a look into an improved handling of voxels affected by partial volume effects.

Overall, DECT-based composition estimation of the most relevant elements was found to be in agreement (within 10% mass fraction) with the ground truth of the samples and would therefore be suitable for use in analytical algorithms or Monte Carlo simulations within a range and/or dose verification workflow in the future.

## Data Availability

The data that support the findings of this study are available upon reasonable request from the authors.
